# Beechwood Creosote in Treatment of Phthisis

**Published:** 1894-03

**Authors:** William J. Cox

**Affiliations:** Flovilla, Ga.


### BEECHWOOD CREOSOTE IN TREATMENT OF PHTHISIS.

BY WILLIAM J. COX, M. D.,

FLOVILLA, GA.

Phthisis is ordinarily considered a disease of the lung characterized by the presence of a peculiar morbid substance called "tubercle."

Its pathology, etiology and symptoms need no discussion, as they are understood by the more intelligent class of physicians.

It is true that the diagnosis is somewhat difficult in the first stages, as there are no physical signs to indicate that the tubercular deposits are being formed, but this should almost lead to a correct diagnosis itself, when there is a history of consumption, with chronic cough and general emaciation.

In the case that I will report I will give the symptoms and physical signs so that my readers may be sure that I was not mistaken in my diagnosis:

Percussion nil, auscultation jerky, wavy inspiration; at end inspiration prolonged and somewhat tubercular, localized in subclavicular region left lung.

The rational signs were, hacking cough, especially in the morning, not relieved by ordinary remedies, progressive emaciation and anemia. Iron and the hypophosphites did no good. A continued elevation of temperature, rapid and weak pulse. Hæmoptysis every two or three weeks. Amenorrhea, and sometimes vicarious menstruation. Vomiting of blood at menstrual epoch. The expectoration was mucous and the eyes pearly and lustrous.

The patient, a young lady aged twenty-nine, had been in somewhat this condition for about two years. Her former physician, an eclectic, had prescribed the various preparations of the hypophosphites with iron and cod liver oil. When I saw her last July she was hardly able to sit up, the emaciation rapidly progressing. She had also been taking large quantities of whisky, which I had her to leave off. Gave her beechwood creosote one drop, with cod liver oil thrice daily, gradually increasing the creosote to six drops per day. For the menstrual trouble I gave her a combination of ponca, aletris and viburnum prunifolium. The cough has about ceased. The menses are regular. She has recovered about twenty pounds in weight and her general appearance is better than ever before. She is still taking the creosote and cod liver oil, which I will have her to keep up for some time, and will report the results to the readers of your journal.

I neglected to mention that there was a distinct history of consumption in the family, the father and one sister having died with it.

She seems now to be on the road to recovery, and I believe she will eventually get well.

---

### PREPARATION OF MERCURIAL PILLS.

Dr. Suinguand (*Pharm. Post*, 1893, XXVI., p. 621) publishes a new practical method of preparing mercurial pills. Thirty grammes (1 oz.) of medicinal soap are dissolved in distilled water, precipitated several times with salt water, well washed out, and finally redissolved in an abundance of distilled water. To this is poured a dilute solution of 13.4 grammes (3⅜ dis.) of mercuric chloride, and the resulting mercury oleate washed by maloxatine. After kneading with powdered licorice root, 100 pills are made from the mass, each of which will contain 15 centigrammes (2¼ gr.) of mercury oleate, or 4 centigrammes of metallic mercury. The pills are finally coated with molten salol. When thus prepared, mercurial pills do not disturb digestion in the least, it is claimed, and are very efficient against all syphilitic symptoms.

